# Sensorized Garments and Textrode-Enabled Measurement Instrumentation for Ambulatory Assessment of the Autonomic Nervous System Response in the ATREC Project

**DOI:** 10.3390/s130708997

**Published:** 2013-07-12

**Authors:** Fernando Seoane, Javier Ferreira, Lorena Alvarez, Ruben Buendia, David Ayllón, Cosme Llerena, Roberto Gil-Pita

**Affiliations:** 1 School of Engineering, University of Borås, SE-50190 Borås, Sweden; 2 School of Technology and Health, Royal Institute of Technology, SE-14152 Stockholm, Sweden; E-Mails: javierfg@kth.se (J.F.); rubenbl@kth.se (R.B.); 3 Department of Signal Theory and Communications, University of Alcala, ES-28871 Madrid, Spain; E-Mails: lap81386@uah.es (L.A.); david.ayllon@uah.es (D.A.) cosme.llerena@uah.es (C.L.); roberto.gil@uah.es (R.G.-P.)

**Keywords:** bioimpedance, GSR, heart rate, mental stress, non-invasive measurement, textile electrodes

## Abstract

Advances in textile materials, technology and miniaturization of electronics for measurement instrumentation has boosted the development of wearable measurement systems. In several projects sensorized garments and non-invasive instrumentation have been integrated to assess on emotional, cognitive responses as well as physical arousal and status of mental stress through the study of the autonomous nervous system. Assessing the mental state of workers under stressful conditions is critical to identify which workers are in the proper state of mind and which are not ready to undertake a mission, which might consequently risk their own life and the lives of others. The project Assessment in Real Time of the Stress in Combatants (ATREC) aims to enable real time assessment of mental stress of the Spanish Armed Forces during military activities using a wearable measurement system containing sensorized garments and textile-enabled non-invasive instrumentation. This work describes the multiparametric sensorized garments and measurement instrumentation implemented in the first phase of the project required to evaluate physiological indicators and recording candidates that can be useful for detection of mental stress. For such purpose different sensorized garments have been constructed: a textrode chest-strap system with six repositionable textrodes, a sensorized glove and an upper-arm strap. The implemented textile-enabled instrumentation contains one skin galvanometer, two temperature sensors for skin and environmental temperature and an impedance pneumographer containing a 1-channel ECG amplifier to record cardiogenic biopotentials. With such combinations of garments and non-invasive measurement devices, a multiparametric wearable measurement system has been implemented able to record the following physiological parameters: heart and respiration rate, skin galvanic response, environmental and peripheral temperature. To ensure the proper functioning of the implemented garments and devices the full series of 12 sets have been functionally tested recording cardiogenic biopotential, thoracic impedance, galvanic skin response and temperature values. The experimental results indicate that the implemented wearable measurement systems operate according to the specifications and are ready to be used for mental stress experiments, which will be executed in the coming phases of the project with dozens of healthy volunteers.

## 1. Introduction

Extensive research efforts and technological advances in textile materials, miniature bioinstrumentation, hybrid microelectronics and wireless communication together with the specific push given by the EC commission supporting several research projects on the theme wearable sensor systems for physiological monitoring such as BIOTEX IST-2004-016789, CONTEXT IST-2004-027291, MERMOTH FP6-IST-508272, MyHeart IST-2002-507816, OFSETH IST-2005-027869, PROETEX IST-2004-026987, STELLA FP6-IST-028086 and HeartCycle FP7-216695 has produced a proliferation of research projects, activities and initiatives within the wearable biomedical sensors and systems [1–4].

In the last decade, several wearable biosensors targeting measurement of different physiological parameters have been used in numerous proof-of-concept projects aiming to implement all kind of applications, ranging from assessment of emotional status [5,6], to monitor activity or patient monitoring. In [3], a comprehensive survey on wearable sensors systems for health monitoring has found more than 20 wearable research prototypes and around 10 commercial wearable non-invasive measurement systems.

Good examples of the use of wearable biomedical sensors technologies for personalized healthcare applications are the sensorized chest strap used to report cardiopulmonary activity status during emergencies [7], the sensorized vest that incorporates fully woven textile sensors to monitor the ECG and respiration rate [8] and the HeartCycle sensorized vest for detection of cardiogenic pulmonary edema [9].

Recent successful examples of wearable measurement technology commercially available include from simple heart rate (HR) monitors for fitness, e.g., the Polar heart rate strap [10], to body worn multiparametric measurement systems, e.g., the EQ02 life monitor manufactured by Equivital [11] or the nECG L1 shirt developed by Nuubo, which is designed to measure the ECG [12].

The availability of such wearable measurement systems fosters the development of personalized health monitoring applications not only for chronic patients and home-care situations but also for personnel working in dangerous conditions.

### 1.1. The ATREC Project

**Figure 1.**
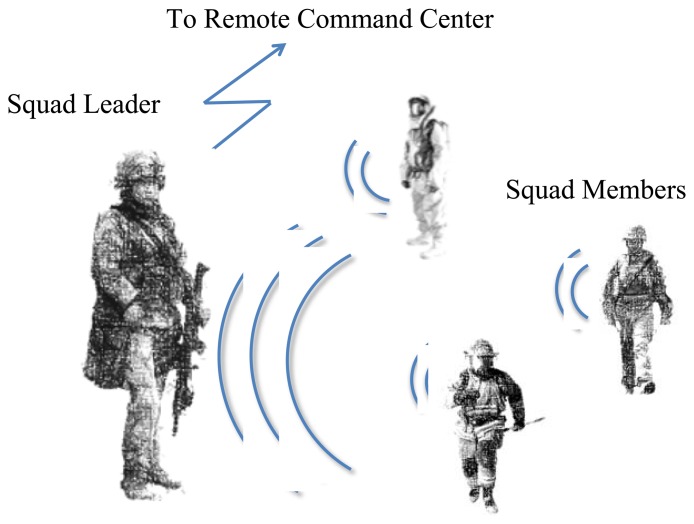
System overview of the full system to be implemented during the ATREC project, which integrates sensorized garments, wireless communication and wearable computing for a real-time assessment. Note that this paper will be focused on the textile-integrated measurement instrumentation portion of the project.

ATREC is the acronym for the Spanish name of the project: Análisis en Tiempo Real del Estrés del Combatiente (Real-Time Analysis of the Stress of the Combatant). The ATREC project aims to set up a complete system to assess the mental stress load of troopers during military activities in real-time, and it is based on the use of wearable measurement systems for the acquisition of non-invasive physiological measurements and using wireless communication; see Figure 1.

In the first phase, the ATREC project aims to identify suitable indicators to assess the autonomic nervous system; to this end, customized wearable instrumentation in sensorized garments has been implemented to perform non-invasive recording of all the above-mentioned physiological variables except EEG activity.

The first phase also includes the use of voice recordings for speech analysis and the use of several types of questionnaires, but these issues are out of the scope of this paper and will be properly described in detail elsewhere. This paper focuses solely on the description of the multi-parameter measurement systems and the sensorized garments implemented for performing an ambulatory study of the activity of the Autonomous Nervous System (ANS). Special emphasis is given to the integration of electrically active textile materials, electronics and regular textile materials.

## 2. Background

### 2.1. Non-Invasive Assessment of Mental Stress

The central nervous system (CNS) comprises the brain and the spinal cord, and through the autonomic nervous system and peripheral innervation of organs and glands, it controls visceral functions that are critical to preserve homeostasis of the organism including the heart's electrical activity, gland secretion, blood pressure and respiration. The ANS is responsible for enabling and controlling the reaction of the body to external and internal stimuli. This control function is performed through the two branches of the ANS: the sympathetic nervous system (SNS) and the parasympathetic nervous system (PNS). Analyzing the activity of the ANS through the SNS and PNS is common practice when assessing stress [13]. Evaluation of ANS activity can be performed by recording and analyzing several physiological variables: heart rate [14], respiration rate (RespR) [15], electroencephalogical (EEG) activity [16], skin galvanic response (GSR) [17,18] and skin temperature [19].

### 2.2. Sensorized Garments and Instrumentation for Assessment of ANS Activity

The available literature indicates that most of the wearable systems made for assessment of ANS activity have targeted the study of emotional, cognitive, physical arousal, mental status and stress [5,6,17,20–28]. Nevertheless several wearable devices and sensorized garments have been implemented and used to record physiological variables to study the response of the ANS during stressful tasks in a non-invasive manner through, e.g., parameters from cardiac activity, GSR dynamics, skin temperature and RespR [15,18,29,30].

The sensorized garments manufactured for recording such relevant physiological variables are mostly chest straps [10,11,31–33] and T-shirts [12,20,21,34–40] aiming to record respiration and heart activity and gloves [20–23], glove-alike [6,24,25] and wrist-bands [26–28,41] for recording GSR and temperature.

Since the turning of century sensorized gloves, gloves-alike and wrist-band have been used for obtaining GSR recordings and performing electrodermal response studies. With the years the level of electronic integration has increased and from using metallic [25–28] or Ag/AgCl external electrodes at the beginning [20,21,24,41] and electrodermal response sensor integrated in the glove later on [23], gloves with textrodes integrated in the garment have been eventually produced [22].

## 3. Multiparametric Wearable Measurement Systems for the ATREC

For the experimental test scheduled under the first phase of the ATREC project 12 sets of wearable measurement devices and sensorized garments have been produced. Each of the sets contains the following measurement elements:

One GSR and Temperature unit, one ECG and Thoracic Impedance recording unit, one Sensorized glove, one upper-arm strap, one chest strap system and six textrodes. Each of these elements is described in detail in the following sections.

### 3.1. Measuring Devices

Two types of devices have been built for this initial phase; one device measures GSR and skin temperature, and the second device records cardiogenic biopotentials and performs impedance plethysmography measurements.

Both devices share some electronic modules such as the microcontroller, the memory module or electrical connections among others. The devices are provided with a 900 mAh lithium-ion battery, and they have an internal 4 GB micro SD memory card to allow long-term recording and off-line analysis.

All the devices are provided with an internal real time clock that it is synchronized with a master unit. The sensor measurements as well as the real time clock are stored in the memory for future recovery and measurement synchronization. The measurements are stored in a micro SD memory card using a FAT32 file system standard. After the measurements are done, the memory card is extracted and a micro SD memory card reader and a computer are used to extract the measurements for storage for later processing.

#### 3.1.1. GSR and Body Temperature

**Figure 2.**
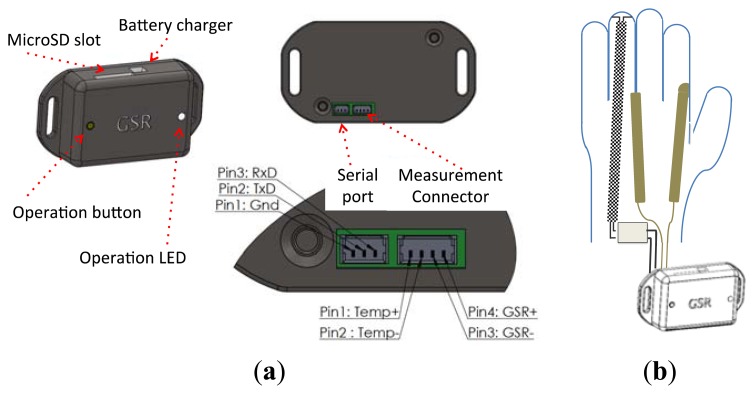
**(a)** Drawings describing the measuring device and its connections. Front view is on the left, back view is on the right and detail of the data and measurement connections is on the bottom. (**b**) Measuring device with a schematic connection to the sensorized glove.

This device enables skin impedance measurements as well as ambient and body temperature measurements. Its compact dimensions, 50 × 35 × 15 mm, allow the device to be integrated in a wearable measurement system. In this case, this device is intended to be used with a sensorized glove or arm-strap that will be described in the following sections; see Figure 2.

**Figure 3.**
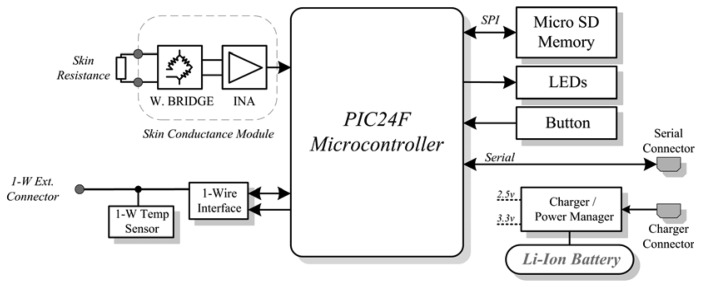
Block diagram of the GSR-Temperature unit.

The GSR-Temperature unit block diagram is depicted in Figure 3, where the main core is a Microchip PIC24FJ64 microcontroller, which contains the firmware that controls the measurement unit. The microcontroller was selected because the low power modes, the suitable number of peripherals that can control and its reduced dimensions. The unit is provided with a button that enables to switch on and off the unit, as well as to initiate the measurement. Two LED displays the status of the unit, such as charging, low voltage or measuring stages.

For measurements of GSR, a Wheatstone-bridge topology with a constant voltage excitation of 0.5 V is used. The voltage is applied to the skin through a resistor of 2 kΩ, which limits the maximum current. The differential voltage at the Wheatstone-bridge is measured by an instrumentation amplifier with the output connected to an analog-to-digital converter (ADC) input on the microcontroller to obtain the voltage that is proportional to the skin resistance value. The GSR is measured with a sampling frequency of 250 Hz.

For the temperature measurements, a 1-wire digital thermometer DS1825 manufactured by Maxim Integrated was used. The 1-Wire® communication protocol allows the use of only one data line and ground to communicate with the microprocessor and also to supply the necessary power to the sensor. The DS1825 incorporates a hardware 4-bit identification code that allows the connection of several temperature sensors to the same data line. The GSR measurement unit is connected to two temperature sensors: one external temperature sensor that is in contact with the skin and one internal temperature sensor placed on the microcontroller board. A sampling frequency of one second is used to sample the temperature readings from both sensors.

#### 3.1.2. ECG Amplifier and EBI Plethysmographer

This device records both ECG signals and the thoracic electrical bioimpedance at a single frequency. The compact dimensions of the device (50 × 35 × 20 mm) make it suitable to be integrated with the chest strap system that will be described in the following section.

**Figure 4.**
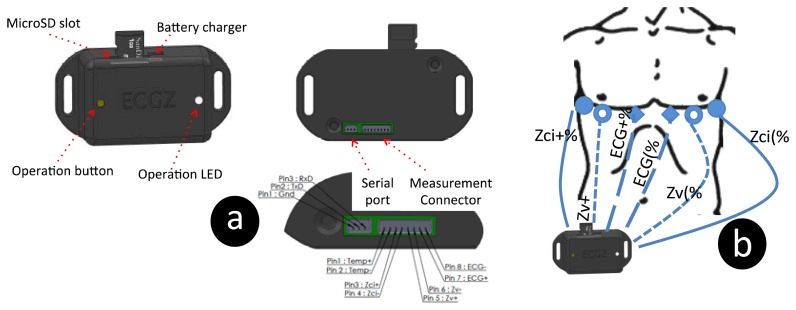
Drawings describing the ECG/ICG device and its connections. (**a**) Front view is on the left, back view is on the right and detail of the data and measurement connections is on the bottom. (**b**) Measuring device with a schematic connection between the ECG and the TEB electrodes placed on the torso for respiration measurements.

The intended use for this ECG/EBI device is to record cardiogenic biopotentials to compute the HR from the obtained ECG and to measure the impedance change caused during breathing to extract the RespR from the recorded TEB signal. In Figure 4b, it is possible to observe the device with a typical electrode connection for ECG and TEB measurements.

**Figure 5.**
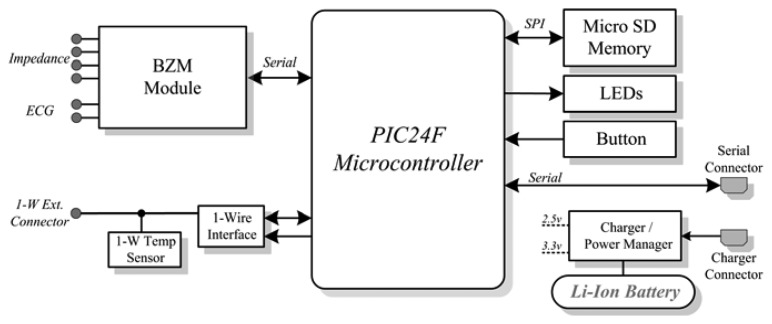
Block diagram of the ECG-Impedance unit.

The ECG-TEB unit block diagram is depicted in Figure 5, where the microcontroller, memory card and other elements are the same as in the GSR-Temperature unit introduced before. The ECG and TEB measurements are performed by the OEM BZM Module manufactured by Z-Health Technologies AB (Borås, Sweden). The BZM module is provided with an internal microcontroller that controls the ECG and Impedance measurements and uses serial ASCII commands to control and transmit the measurements to the main microcontroller.

The ECG signal is measured by a 1-lead instrumentation amplifier topology suggested by Merritt *et al.*[42]. The biopotential signal is sampled by the BZM unit at a sampling frequency of 250 Hz. The TEB measurements are obtained using an excitation frequency of 50 kHz and a sampling frequency of 100 Hz. The impedance estimation core implemented in the BZM unit is based on the SoC AD5933 manufactured by Analog Devices Inc. (Norwood, MA, U.S.A.) by implementing an analog front-end customized for 4-electrode measurements [43].

### 3.2. Sensorized Garments and Textrodes

The following garments have been constructed with several types of textile materials including conductive fabrics for the textrodes and electrical connections. The conductive fabric used is the Shieldex® Fabric P130+B manufactured by STATEX Gmbh (Bremen, Germany). All the sensorized garments and their different supporting instrumentation elements mentioned in the previous section are described in this section.

#### 3.2.1. Glove for GSR and Skin Temperature

**Figure 6.**
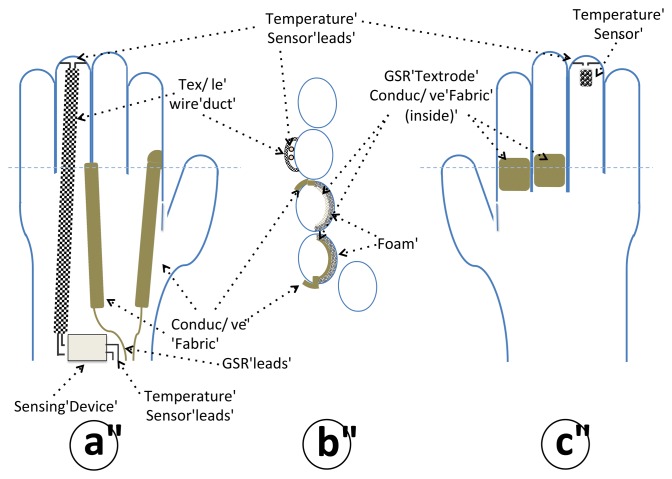
Schematic of the sensorized glove. (**a**) Upper view of the glove. (**b**) Cross-sectional view of the glove at the proximal phalanx in a perpendicular plane to the palm. (**c**) Palm view.

Two textile electrodes were integrated into the proximal phalanx of the index and middle fingers on the inside of the glove to measure the GSR, and a temperature sensor was placed in the tip of the ring finger of the glove to sense the peripheral skin temperature; see Figure 6 for details.

**Figure 7.**
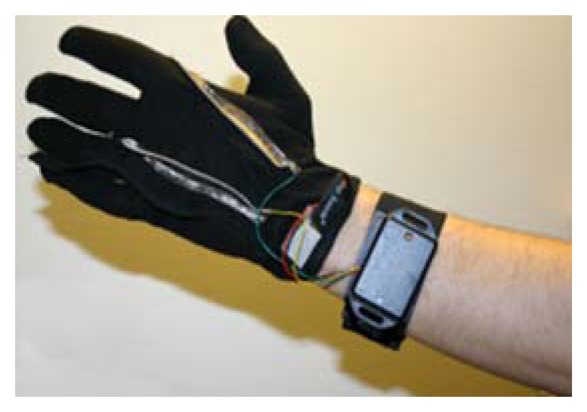
Sensorized glove connected to the measuring unit, which is fastened to the wristband.

The textrodes and the temperature sensors are connected via four cables with the measuring device, which is fastened with Velcro to a wristband as shown in Figure 7.

#### 3.2.2. Upper-Arm Strap for GSR and Skin Temperature

**Figure 8.**
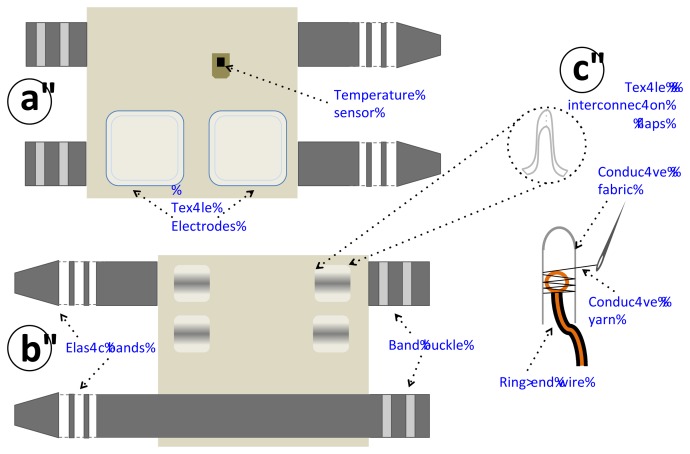
Schematic of the constructed sensorized upper arm strap. (**a**) Inside view showing the sensors. (**b**) Outside view where the sensing device has been placed and connected. (**c**) Detail of the textile-electronic interconnection, which is achieved by sewing conductive fabric through a ring-shaped end using conductive yarn.

**Figure 9.**
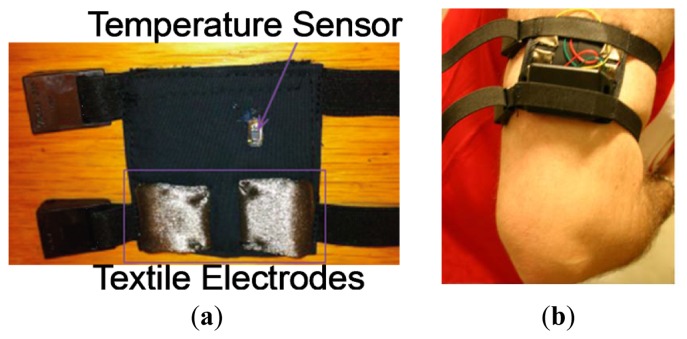
**(a)** Constructed upper arm strap. (**b**) Measuring device and strap worn on the upper arm.

An upper arm strap with two integrated textile electrodes was constructed to sense the galvanic skin response between the electrodes on the skin's surface. A DS1825 sensor was also integrated in the inner lining of the strap to contact the skin; this method measures the superficial skin temperature. Figure 8 shows the design of the constructed sensorized garment and Figure 9a shows the upper arm strap. Figure 9b shows the sensorized arm strap connected to the measurement unit for GSR and body temperature measurements and worn on the arm.

#### 3.2.3. Chest Strap System for Cardiac and Respiration Recordings

**Figure 10.**
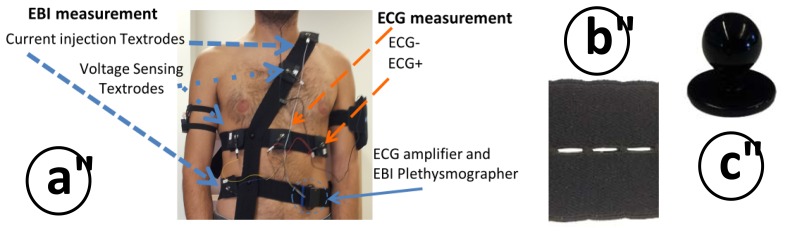
**(a)** Chest strap system shown with placement of ECG and TEB electrodes. (**b**) Detail of the elastic perforated band. (**c**) Fixation between straps was accomplished by a chef jacket button.

A chest strap garment with repositionable textile electrodes was developed to record 1-lead ECG measurements from 2 textrodes and tetrapolar TEB measurements from four other textrodes. The possibility of placing the electrodes in any place along the horizontal and vertical straps enables different types of TEB measurements to be performed; see Figure 10a. Depending on the placement locations of the textrodes around the surface of the thorax and abdomen, the TEB measurement will have relatively more or less cardiac and respiratory components, which allows us to perform a multi-parametric signal recording if required.

Each strap is made of a highly elastic band with 1 cm perforations every 2 mm; see Figure 10b. These perforations enable the repositioning of the textrodes along the straps, and fixation points between straps are fastened with chef jacket buttons; see Figure 10c.

#### 3.2.4. Repositionable Textrodes

**Figure 11.**
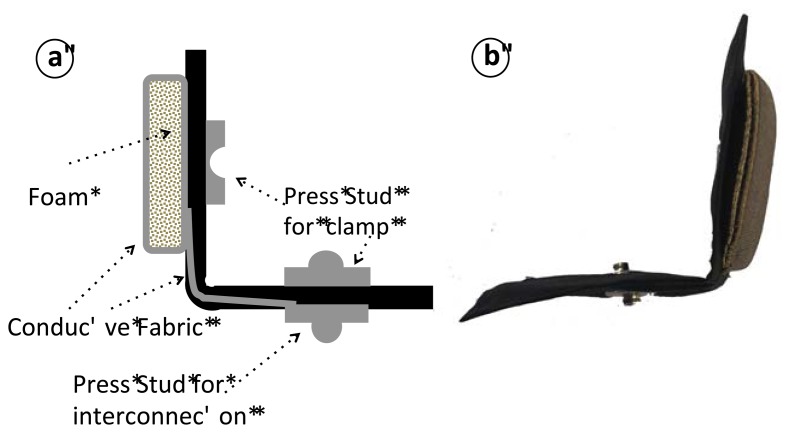
**(a)** Schematic of the repositionable textrode. Note that when the electrode is folded, the male and female press studs will be clamped. (b) Constructed repositionable textrode.

The textile electrodes are square with side length of approximate 5 cm producing a surface area of 25 cm^2^ and are fashioned as a textile structure that is folded over itself and creates a clamp through a male-female pair of press studs; see Figure 11. The connection between the measurement leads and the textrodes are achieved through a male press stud.

## 4. Measurement Results

A total number of 12 devices and sensorized garments for each modality have been produced and the correct operation of the measuring devices and sensorized garments has been tested at least on two different volunteers obtaining at least 2 minutes of GSR recordings with the glove and the arm strap as well as ECG and respiration activity. This section presents a set of measurements for a single subject obtained during those correct operation tests with a measurement device garment of each kind. More measurements recorded with three different measurement sets are available in Appendixes A and B.

### 4.1. GSR and Temperature

**Figure 12.**
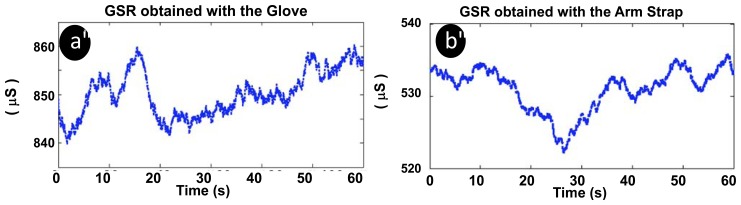
Galvanic skin response measurements. (**a**) Glove. (**b**) Upper arm strap.

Skin temperature and GSR were recorded in several subjects. The skin temperature measurements were very stable approximately 34 °C, and both garments produced flat recordings. However, the GSR measurements exhibited larger variance; see Figure 12. Note that the GSR data have been filtered with a moving average window of 5 seconds as in [44].

### 4.2. Thoracic Measurements

#### 4.2.1. ECG Recordings

**Figure 13.**
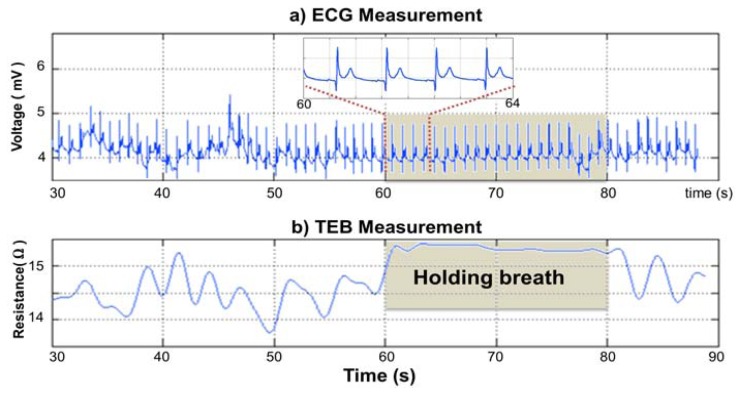
**(a)** ECG recording. (**b**) Transthoracic electrical bioimpedance recording.

Surface cardiac biopotentials were recorded in several subjects with the wearable measurement system presented in Figure 10a. The ECG recording plotted in Figure 13a shows 60 s of recorded ECG. As shown in the 4 s zoomed segment, the recording is very fair and allows for direct detection of the R complex for a heart rate assessment. The first 25–30 s show slow activity related to respiratory movement. Between 60 and 80 s, the subject held his or her breath; it is possible to see that the ECG recording was completely stable.

#### 4.2.2. Thoracic EBI Recordings

TEB recordings were obtained simultaneously with the ECG recording (see Figure 13) using the wearable measurement system presented in Figure 8a for several subjects. A 60 s segment of the TEB recording is shown in Figure 13b. The respiration activity is easily observable in this plot as the resistance increases and decreases according to the respiration cycle. Note that the changes stop between 60 and 80 s on the plot while the subject was holding his or her breath.

## 5. Discussion

### 5.1. Multiparameteric Wearable Textrode-Enabled Measurement System

In this work textrode-enabled sensorized garments with wearable measurement devices for performing multiparametric non-invasive measurements have been implemented and replicated in series of 12. The intended goal of such wearable instrumentation is to identify which parameters might be more useful for assessing ANS activity as a potentially precursor for mental stress and workload.

As mentioned at the introductory sections of this work, several wearable measurement systems have been implemented for numerous different applications [1–4,7–9,15,18,20–23,29,30], especially in the last decade as a result of the aforementioned projects funded by the EC commission. Although the main application of this wearable instrumentation is to be used in an ambulatory study of mental stress and workload, this manuscript aims to emphasize the description of the sensorized garments, textrode interconnection and the textile-electronic integration techniques used to implement the wearable measurement systems leaving the application of assessment of mental stress in secondary plane.

Apparently for reasons beyond our knowledge, in a significant portion of the literature available on this topic, most of the articles focus primarily on the application, describing the wearable instrumentation and sensorized garments in relatively shallow manner. See any of the following references [1–4,7–9,15,18,20–23,29,30]. In this work the focus is centered on the sensorized garments and the wearable instrumentation, and issues related to its design, use and production. Therefore the use of experimental recordings has been intended to show the proper functioning of the multiparametric and non-invasive wearable instrumentation implemented in the project.

Information related to the execution of tests and experiments targeting the assessment of mental stress and mental workload performed in dozens of volunteers completing pre-defined specific tasks will be reported once the studies have been finalized elsewhere.

### 5.2. Different Locations for GSR Measurements

The two different sensorized garments that we constructed allow the evaluation of which of the different positions is best for performing GSR recordings both at rest and during physical activity. By considering *a priori* the high level of activity of the hands during normal and combat activities, it is likely that such recordings might be prone to all sorts of movement artifacts. Because the finger is considered among the most suitable locations for GSR sensing, we will not be able to confirm which of the locations is most suited for performing robust GSR recordings until the end of the experimental phase.

### 5.3. Placement of Textrodes on the Thorax

The many possible locations given by the clamping textrodes and perforated elastic bands allow us to explore the best location to obtain ECG recordings. Moreover, they also open up the possibility to place TEB electrodes in positions where they can record not only respiration activity but also cardiac activity. If such dual-use of the TEB electrodes is successful, it would be possible to extract the HR not only from the ECG measurements but also from TEB recordings [45], which would contribute to a more robust system.

### 5.4. Textile-Electronic Interconnections

To construct these sensorized garments, two simple but robust approaches have been used to interconnect conductive textile elements with metallic wires and contacts. The use of a press-stud through the conductive fabric is the most-used approach to perform such types of textile-electronic interconnection, and it has been observed in commercial products [10,12] as well as in research prototypes [46,47]. Sewing conductive yarn through conductive fabrics has also been used before to establish an electrical connection [48]. To interconnect metallic wires with conductive textiles in our garment, sewing with conductive yarn through a ring-terminated wire placed between two pieces of conductive fabric was used. Because the fabrics are very elastic, the resulting interconnection is both tight and mechanically robust.

### 5.5. Silver on Textrodes

The conductive material in the textile fabrics that we used to construct the textrodes is high-grade silver. According to the recommendation that any medical device containing silver in contact with the skin should be classified as a Class III medical device [49], it would convenient for cases in which the Medical Device Directive [50] applies, to explore the use of alternative conductive textile materials that do not contain silver such as polymer electrodes [51].

### 5.6. Size of the Wearable Systems

The size of the implemented devices and their lightweight allow for the devices to be worn together with the garments and this way the systems can be used for ambulatory measurements. Although a smaller size could be achieved from the electronic design perspective, the size of the devices in this case is basically set by the size of the batteries, required to enable the performance of the different type of test and experiments.

## 6. Conclusions and Outlook

The proper functioning of the implemented series of sensorized garments and measurement devices allows us to conclude that the produced wearable systems are ready for the next stage of the ATREC project: The experimental phase. Such phase is focused on the data recording in volunteers performing activities specifically designed to cause stress. The experimental phase is being executed as expected and measurements have been performed on more that 50 volunteers with the assembled sensorized garments, and the implemented wearable measurement instrumentation. The obtained recording and their analysis will be reported at the end of the experimental phase.

## Figures and Tables

**Figure A1.**
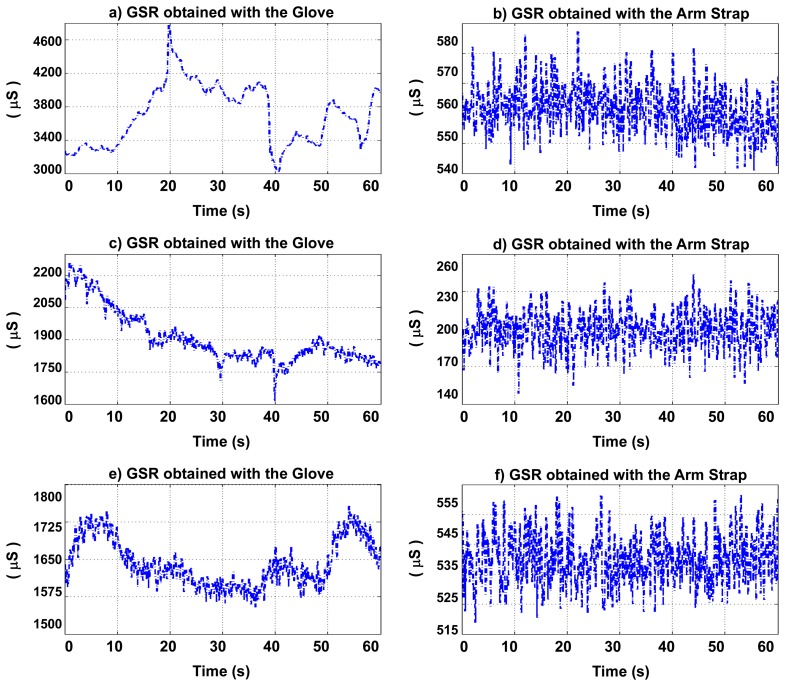
Galvanic skin response measurements. (**a**), (**c**) and (**e**) obtained with the glove and (**b**), (**d**) and (**f**) obtained with the upper arm strap.

**Figure B1.**
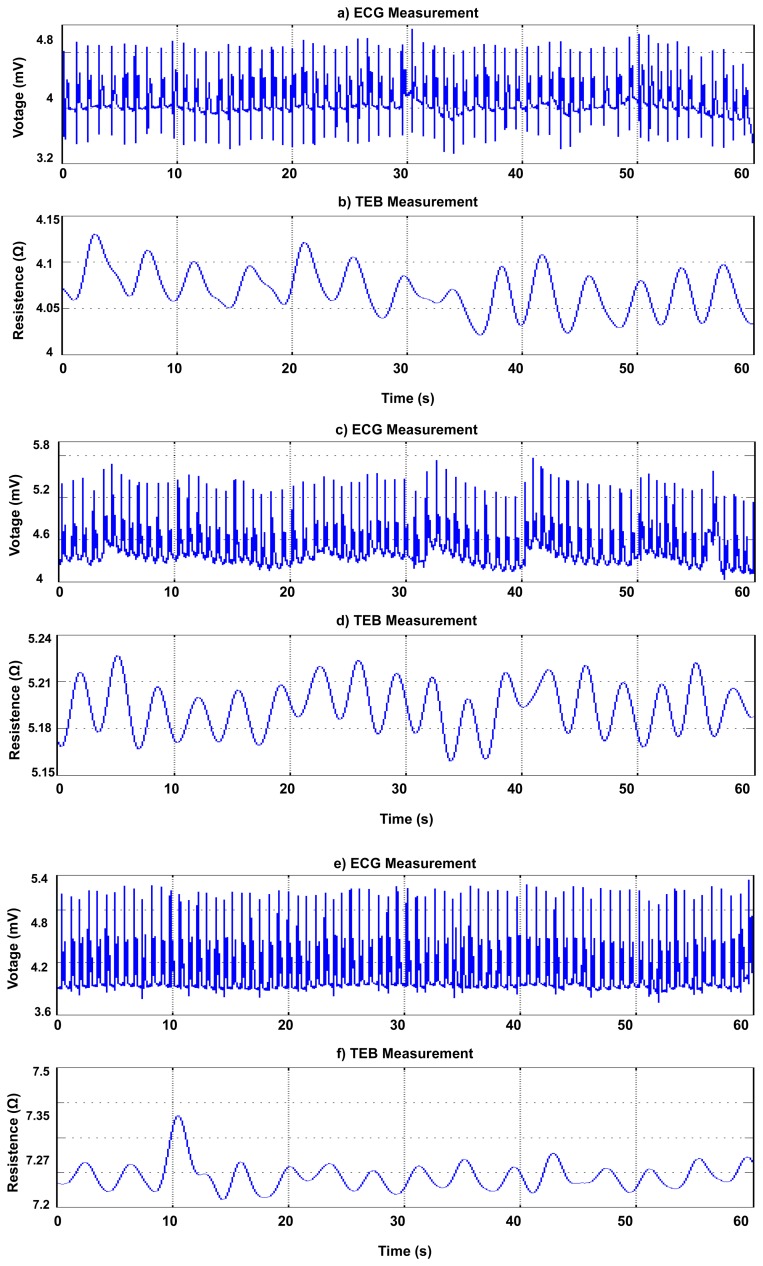
ECG measurements in (**a**), (**c**) and (**e**). TEB measurements in (**b**), (**d**) and (**f**).
